# Label-Free Toxin Detection by Means of Time-Resolved Electrochemical Impedance Spectroscopy

**DOI:** 10.3390/s100100655

**Published:** 2010-01-18

**Authors:** Changhoon Chai, Paul Takhistov

**Affiliations:** School of Environmental and Biological Sciences, Rutgers, the State University of New Jersey, New Brunswick, NJ 08901, USA; E-Mail: chchai@rutgers.edu

**Keywords:** *Staphylococcus* enterotoxin B, electrochemical impedance, nano-porous aluminum, antibody immobilization, immunoreaction

## Abstract

The real-time detection of trace concentrations of biological toxins requires significant improvement of the detection methods from those reported in the literature. To develop a highly sensitive and selective detection device it is necessary to determine the optimal measuring conditions for the electrochemical sensor in three domains: time, frequency and polarization potential. In this work we utilized a time-resolved electrochemical impedance spectroscopy for the detection of trace concentrations of Staphylococcus enterotoxin B (SEB). An anti-SEB antibody has been attached to the nano-porous aluminum surface using 3-aminopropyltriethoxysilane/glutaraldehyde coupling system. This immobilization method allows fabrication of a highly reproducible and stable sensing device. Using developed immobilization procedure and optimized detection regime, it is possible to determine the presence of SEB at the levels as low as 10 pg/mL in 15 minutes.

## 1. Introduction

The recent advances in electroanalytical chemistry provide a new opportunity for the development of biosensors. These electrochemical devices are relatively inexpensive and well suited for miniaturization, which is critical for field-deployable applications. Significant progress in the development of electrochemical affinity-based biosensors (immunosensors) has been shown with various electrochemical techniques for the detection of DNA [1,2] and proteins/toxins [3–8].

Electrochemical impedance spectroscopy (EIS) is a highly effective analytical method to characterize physico-chemical properties of the electrode/analyte interface [9]. EIS is a sensitive, non-destructive technique suitable for monitoring the dynamics of bound and/or mobile charges near the sensor’s surface [10–13]. If EIS is used in the sensor’s signal transducing system, the detection of target molecules can be accomplished directly without labeling [14]. However, the development of reliable EIS-based sensors for the detection of low concentrations of biological toxins remains difficult due to poor understanding of electrochemical processes at the sensor/sample interface. Traditional factors that determine sensor’s output signal (*i.e.,* charge transfer resistance and electrical double layer capacitance) can not be interpreted correctly when concentration of antibodies (Ab) is low and surface coverage by the antibody/antigen (Ab/Ag) complexes is far from its maximum value [15].

Staphylococcal enterotoxin B (SEB) is an exotoxin produced by *Staphylococcus aureus*. It causes food poisoning in humans and is classified as a Category B bioterrorism agent. Due to its high virulence and extremely low lethal dose [16], there is a strong need to develop a rapid label-free method for the detection of trace concentrations of the toxin. In the past years, a number of studies had been carried out employing conventional techniques for SEB detection, including chromatography, enzyme-linked immunoassay (ELISA), magnetic microplate chemifluorimmunoassay (MMCIA), and surface plasmon resonance (SPR) [17,18]. However, these methods are complex, require relatively expensive equipment, materials, and highly trained personnel.

## 2. Results and Discussion

### 2.1. Immunosensor fabrication and anti-SEB immobilization

Surface morphology of the sensor’s substrate is crucial for the electrochemical immunosensor development since it affects Abs’ immobilization, their spatial arrangement and local distribution of the current density at the sensor’s surface. To increase surface area and holding capacity of the sensor’s substrate, we have applied an electrochemical nano-patterning (anodization) of aluminum—a well-proven technique for surface treatment and improvement of aluminum materials. Before the anodization, an uneven surface of cold-rolled aluminum disk was electropolished to normalize its surface morphology. At the optimal regime (42 V, 40 s, at 4 °C), the aluminum surface becomes smooth and covered with well-ordered linear patterns of 3–5 nm in depth [19]. Subsequent anodisation of electropolished aluminum substrate in 0.3 M oxalic acid results in the formation of a well-ordered nano-porous surface structure. After the pore widening step (5% w/v H_3_PO_4_), the diameter of nano-pores becomes ∼60–80 nm.

**Figure 1.**
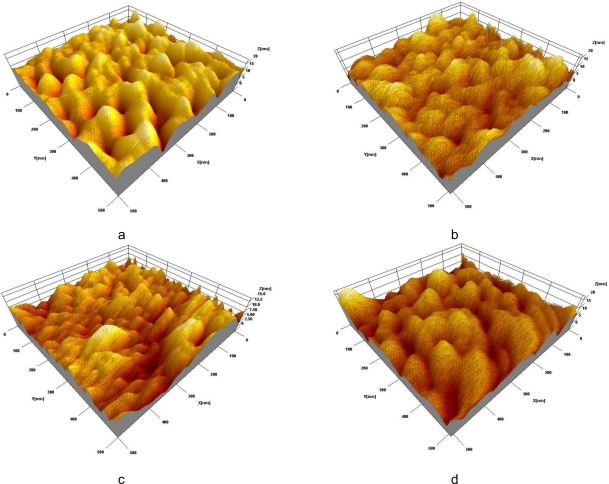
SPM images of a nano-porous aluminum surface: silanized with APTES (a); with anti-SEB immobilized (b); empty sites blocked with ethanolamine (c); morphology changes due to immunoreaction (d).

For the Ab immobilization, a fabricated nano-patterned substrate was modified with the APTES (see Figure 1a) as described in the literature [20]. There are several advantages to use an APTES for the surface activation: it has both a non-hydrolysable hydroxyl group, which is highly reactive to the metal oxides and amine group that presents a reactive moiety, enabling strong interaction with corresponding Ab amino group [21]. Anti-SEB was covalently bonded on the silanized aluminum surface in the presence of glutaraldehyde, which resulted in the formation of well-recognizable surface structures of various Ab aggregates (see Figure 1b). After successful Ab immobilization, the sensor’s surface has been treated with 100 mM ethanolamine solution for 1 hr to block remaining vacant sites and fix attached antibodies. Figure 1c clearly indicates changes of the surface morphology due to ethanolamine adsorption. Large gaps between the Ab clusters disappear and the surface becomes smooth and uniform. For the control purpose, developed sensor was exposed to the 0.3% NaCl solution containing 10 μg/mL of SEB for 60 min. SPM image of the sensor’s surface shows development of new structures on the surfaces (see Figure 1d). These surface morphology changes are due to the development of Ab/Ag immuno-complexes.

### 2.2. SEB detection and optimization of measuring parameters

The real-time detection of SEB trace concentrations in the samples with high variability of the sample matrix properties (e.g. clinical samples, foodstuff) requires significant improvement of the detection methods from those reported in the literature [22]. To develop a highly sensitive and selective detection technique it is necessary to determine the optimal measuring conditions for the sensibilized electrode (*i.e.*, sensor) in three domains: time, frequency and polarization potential. To optimize measuring conditions in order to obtain the best possible performance of a biosensor, a multi-step design approach is required.The steps include:

- To study electrode dynamics associated with the immunoreaction in the presence of excessive amount of highly conductive background electrolyte. The complete electrochemical behavior of a system can be investigated by sweeping the electrode potential with time and recording the resulting current as a function of potential. In that case, cyclic voltammetry is used to identify polarization regime of anti-SEB/SEB reaction and corresponding electrode charge transfer resistance. It allows to determine the optimal detection conditions with the highest sensitivity and stability of immobilized anti-SEB.
- To use time resolved electrochemical impedance spectroscopy (TREIS) to identify the perturbation frequency that corresponds to the specific interaction of probing charge carriers with Ab/Ag complexes. Measuring the sensor’s impedance at this optimal frequency provides the best signal-to-noise ratio and stability of an output signal.
- To determine the dynamic characteristics of detector and to identify the minimal detection time of the sensor by recording sensor’s complex impedance at the optimal single frequency as a function of time.

### 2.3. Cyclic voltammetry of the SEB immunosensor

Cyclic volatammetry (CV) is a powerful technique to characterize the chemical composition and surface morphology of an electrode surface that widely used to investigate the surface-associated electrode processes [23]. CV responds sensitively to deposition of organic substances on the electrode surface as well as to the changes due to development of new organic structures as a result of Ab/Ag reaction [5,24–26]. Cyclic voltammetry data provide crucial information for the correct interpretation of observed changes in surface properties. It allows to distinguish the changes induced by an Ab/Ag reaction form the changes due to a non-specific interaction of the background electrolyte with the sensor surface. We perform all measurements in two parallel experiments in two different media: one in the pH 6.0 0.3% NaCl with pre-determined concentrations of SEB (10 μg/mL), and the other in the same medium with no SEB added.

**Figure 2.**
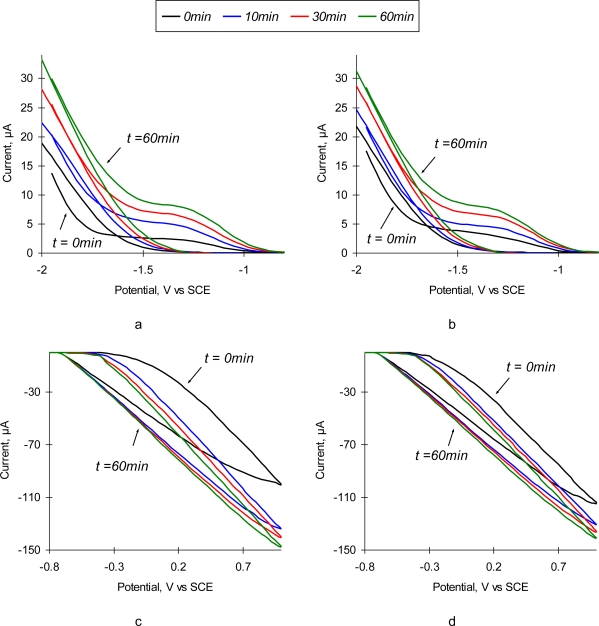
Cyclic voltammogramms of SEB immunosensors in 0.3% NaCl solution without toxin (a,c) and with addition of 10 μg/mL SEB (b,d) as a function of time (0 min, 10 min, 30 min, and 60 min).

Figure 2 depicts the resulting plots of cyclic voltammetry study of SEB sensors in the solution containing 10 μg/mL of SEB and corresponding control solution with no toxin added. Due to the significant difference in the scale, cathodic and anodic branches of the voltammogramm are shown separately. As one can observe, the change of charge transfer resistance of the positively polarized electrode (Figure 2c) is much higher than that of the negatively polarized one (Figure 2a). The resulting shift in the anodic current is about 50 μA (Figure 2a), and only ∼20 μA for the cathodic current (Figure 2c). Addition of SEB into the solution does not produce significant changes in the shape of the cyclic voltammogramms (Figure 2b,d). There are very small differences in the observed currents in both solutions. However, time dependence of CVA in SEB solution is profoundly different. The slope of the anodic branch does not change gradually (Figure 2d) as in pure NaCl solution (Figure 2c). Rapid changes during the first 20 min slow down at longer exposure times (Figures 2c,d). This behavior is in a good agreement with the reaction kinetics and characteristic times for diffusion-controlled immunoreactions [27].

Observed CVA shapes are typical for the diffusion-controlled processes, with no profound peaks commonly existing in red/ox reactions. It is important to note that the value of hysteresis between the ascending and descending parts of CV scan remains the same during the experiment. One can assume that all current is carried out by the background electrolyte ions, and redistribution of the target analyte (*i.e.*, Ag) in the solution is not impacted by electromigration of the current carriers.

It seems that the optimal value of polarizing potential at the sensing surface, which allows to reveal the formation of Ab/Ag complexes, is ∼1 V. However, the results of performed chronopotentiometry experiments with a sensor polarized at 1 V and consequent SPM imaging of the surface (data not shown) indicate that the high values of positive polarization potential cause detachment of immobilized Ab from the sensor’s surface. This may happen due to replacement of Ab by the negatively charged ions from the solution. We have found experimentally that the optimal polarization voltage, which allows the precise detection of the immunoreaction but does not cause Ab detachment, is 0.1 V *vs.* Ag/AgCl electrode. It is noticeable that in both solutions the point of zero charge (isoelectric point) of the sensor’s surface is −0.765 V, which corresponds to the isoelectric point of pure aluminum [28]. Thus, one can conclude that the ions of a background electrolyte penetrate through the immobilized Ab layer and determine the equilibrium potential of the sensor’s surface.

### 2.4. Time-resolved electrochemical impedance spectroscopy

Based on the results of CV experiments, we can quantify the conditions for EIS detection schemes. The use of a non-polarized electrode is very attractive for future field applications, since the schematics is very simple. Additionally, a 2-electrode measuring scheme provides fewer disturbances to the surface composition of the sensor by probing surface impedance near the equilibrium potential. On the other hand, the polarization of a working electrode allows to achieve better sensitivity of the sensor due to optimization of the charge transfer at the sensor’s interface.

The measuring parameter in TREIS is impedance *Z*(*t, ω*), a complex resistance that can be represented as:

$$\dot{Z}(t,\;\omega )=\frac{U(t,\;\omega )}{I(t,\;\omega )}=Z_{0}\;(t)e^{j\theta }=Z^{\prime }\;(t,\;\omega )+jZ^{\prime \prime }\;(t,\;\omega )$$

To compare output signals obtained from different sensors in various experimental conditions, we use normalized values of the real and imaginary parts of a complex impedance:

$$Z_{\mathrm{norm}}(t,\;\omega )=\frac{Z(t,\;\omega )}{Z(0,\;\omega )}$$

The normalization of obtained data allows discovering the detection patterns that would be undetectable with the standard data representation. EIS experiments were designed in the same manner as cyclic voltammetry studies. Two sets of measurements, one in a pure background electrolyte, and the other with SEB added to the medium, were performed.

#### 2.4.1. TREIS analysis of non-polarized SEB immunosensor

**Figure 3.**
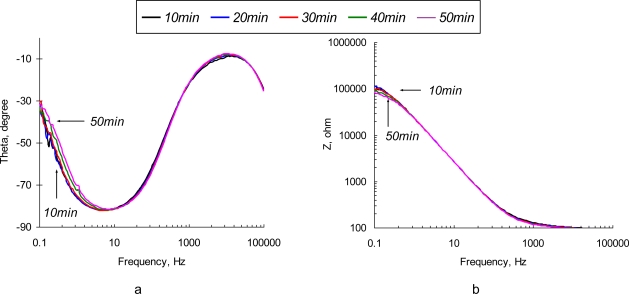
Phase angle (a) and impedance spectrum (b) of a non-polarized (*φ_eq_* = −0.765 V *vs.* Ag/AgC) SEB immunosensor in 10 μg/mL of SEB in 0.3% NaCl solution.

Typical impedance spectra obtained with the non-polarized SEB sensor are depicted in Figure 3. Observed changes in the impedance value and phase angle are associated with two major effects: adsorption of solute ions onto the electrode surface and protein layer and formation of the Ab/Ag complexes (immunoreaction). The most significant changes of both impedance and phase angle are observed at low (<1 kHz) frequencies. It is clear that the impedance of the sensor drops tremendously as probing frequency increases. Recognizable deviations in the impedance value are observed in the high frequency region.

**Figure 4.**
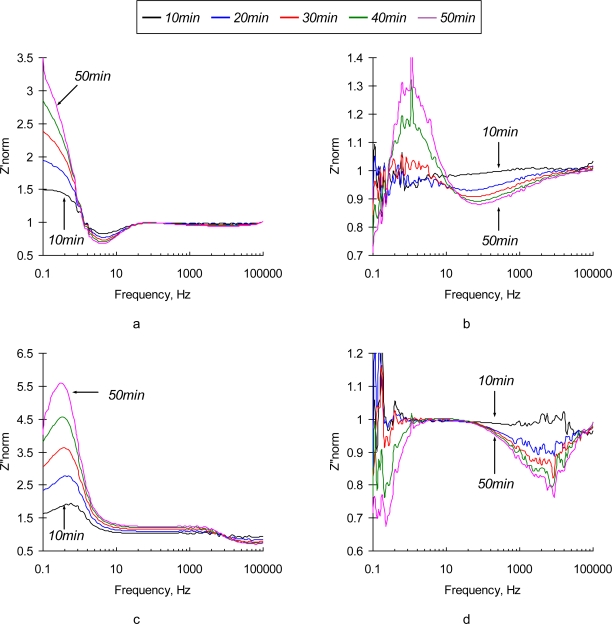
Normalized values of *Z′* (a,b) and *Z″* (c,d) parts of electrochemical impedance spectra of a non-polarized (*φ*= −0.765 V *vs.* Ag/AgCl) SEB immunosensor in 0.3% NaCl (a, c) and 0.3% NaCl + 10 μg/mL (b, d) as a function of time (10 min, 20 min, 30 min, 40 min, and 50 min).

Normalized values of real and imaginary parts of the complex EI spectra are depicted in Figure 4. It is not surprising that the most significant relative changes in phase angle values are observed at the low frequencies (<10 Hz). Electric field-enforced ionic transport directly interferes with the Ab adsorption-desorption process at the surface. Additionally, the characteristic rate constant of the immunoreaction is ∼1.0 × 10^−9^ M^−1^ [29], which gives us characteristic frequency values in the same range. The phase angle spectra of the sensors exposed to the pure NaCl and SEB-containing solutions are similar in shape, although the maximum on the phase angle curve for SEB-containing medium is shifted towards the high frequency region (see Figure 4d). This frequency shift is quite small and is unlikely to be used for the detection purposes. The real parts of impedance spectra of pure NaCl and SEB-containing solutions that are depicted in Figures 4a,b have significantly different patterns in time and frequency domains. An increase in low frequency resistance of the sensor surface in pure NaCl solution is followed by its decrease at high frequencies (Figure 4a). These two regions, therefore correspond to the surface layers that differ in spatial arrangement and transport properties. Addition of SEB toxin causes dramatic changes in the surface response (Figure 4b). A drop in low frequency resistance and appearance of characteristic peaks at 30−50 kHz are the results of immunoreaction. These patterns are distinct and reveal the unique signature of a target analyte. However, due to the extremely low impedance values, signal-to-noise ratio of the sensor in this frequency range is far from desirable, which limits application of the non-polarized sensor electrodes for relatively high concentrations of the toxin (∼0.5 μg/mL).

#### 2.4.2. TREIS analysis of polarized SEB immunosensor

**Figure 5.**
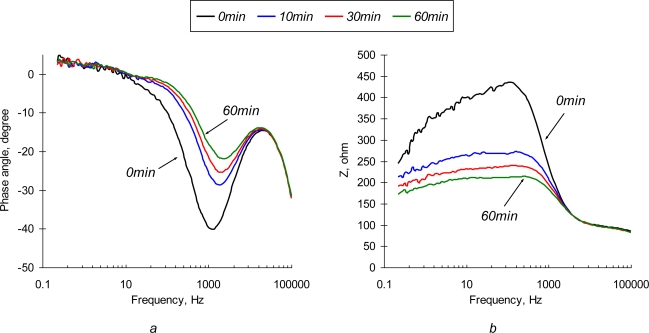
Phase angle and impedance spectrum of polarized (Δ*φ* = +0.1 V *vs.* Ag/AgCl) SEB immunosensor in 10 μg/mL of SEB in 0.3% NaCl solution.

To enhance the sensor’s sensitivity one should choose the measuring conditions so that a small perturbation would cause the most significant changes in the charge transfer at the sensor interface. This regime is characterized by the highest values of the hysteresis of the CVA curves. Based on the results from CV analysis, immunosensibilized electrode was polarized at +0.1 V vs Ag/AgCl. This regime provides the maximum difference between polarization and depolarization electron transfer rate, at the sensor’s interface. As shown in Figure 5, polarization of the sensing element results in dramatic improvements of the sensor’s response over the frequency domain,. Time-dependent changes in phase angle and impedance are much more pronounced than those of a non-polarized electrode. The shapes of the spectra change significantly. The impedance value decreases as perturbation signal changes from low to high frequency, similar to the non-polarized electrode.

Significant changes in the impedance and phase angle are observed at low (<1 kHz) frequencies. A decrease of impedance in the low frequency region is found in TREI spectra of 0.3% NaCl solution and SEB contaminated medium. Significant changes in the impedance in the low frequency domain are dominated by the adsorption/desorption interactions of a background electrolyte with the immobilized Ab layer and sensor’s substrate.

**Figure 6.**
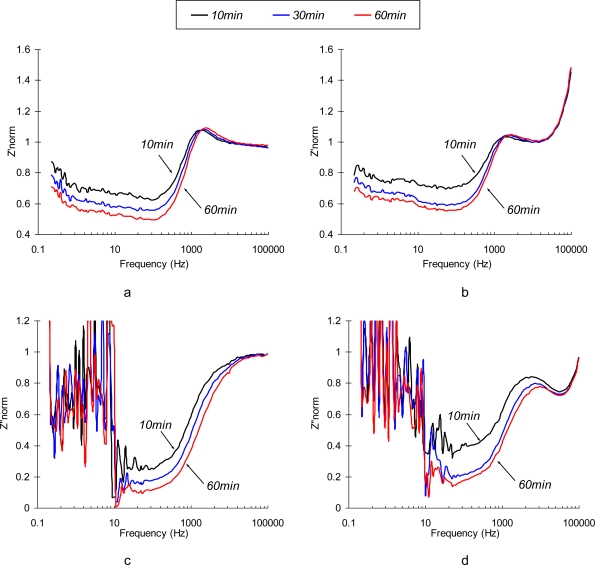
Normalized values of *Z’*(a,b) and *Z”*(c,d) parts of the electrochemical impedance spectrum of a SEB immunosensor polarized at Δ*φ* = +0.1 V *vs.* Ag/AgCl in 0.3% NaCl (a,c) and 0.3% NaCl +10 g/mL SEB (b,d) media as a function of time (10 min, 30 min and 60 min).

Normalized real and imaginary parts of immunosensor’s response in the described experiments are depicted in Figure 6. The behavior of polarized immunosensors in a low frequency region is very similar in both media. At the frequencies that correspond to the characteristic reaction time (<10 Hz), the spectrum loses its monotonicity and exibits irregular peaks due to an overlap of the characteristic times of perturbation signal and immunoreaction (see Figure 6c,d).

The major difference between the sensor’s responses in SEB contaminated and control samples is observed in the medium frequency region (∼10 kHz). This finding is quite reasonable since an additional surface layer existing due to the formation of Ab/Ag complexes, is very thin, and can only impact migration of ions at high frequencies, where the characteristic migration time corresponds to the relaxation time in the newly formed SEB Ab/Ag layer. The optimal frequency range has to be determined between 10∼50 kHz, where the effect of supporting electrolyte is negligible, but the changes of impedance with time (following diffusion-controlled kinetics of immunoreaction) by Ab/Ag layer are significant. Additional criteria are also important in choosing the optimal detection frequency: low signal-to-noise ratio, signal stability, and reproducibility of the detection results. We used a multi-variable optimization (data not shown) to identify the best possible detection frequency for high-performance immunosensors, and if has been determined to be 31 kHz.

### 2.5. Detection of ultra-low SEB concentrations with polarized immunosensor

**Figure 7.**
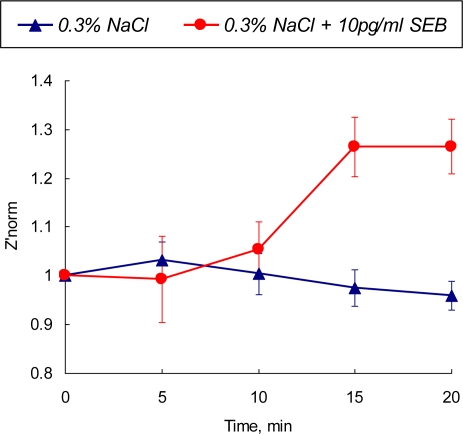
Normalized value of *Z′_norm_* at a single frequency (31 kHz) electrochemical impedance of a polarized SEB immunosensor in 0.3% NaCl and 0.3% NaCl + 10 pg/mL SEB media as a function of time.

The optimal parameters for toxin detection have been identified from the cyclic voltammetry experiments and time-resolved EIS data as follows: the detection frequency of 31 kHz and polarizing potential of +0.1 V vs Ag/AgCl. The real part of SEB biosensor impedance was analyzed at the identified optimal conditions. The normalized values of the real part of impedance in 0.3% NaCl solution containing 10 pg·mL^−1^ are depicted in Figure 7 and compared with the values obtained for the control (no SEB added) system.

Performed 3-D parametric optimization of the detection method (polarization value, frequency domain and detection time) expands the detection limit of the developed biosensor up to picogram quantities of the toxin. A statistically recognizable difference in the sensor’s output appear after 10 min of exposure then reaches the saturation level after 15 min of contact with the tested sample. The output signal is stable and there is no overlap between SEB-contaminated and control media

## 3. Conclusions

In this work, the design of an impedimetric immunosensor for rapid and label-free detection of trace concentrations of *Staphylococcus* enterotoxin B is presented. The developed biosensor combines an antibody as a molecular recognition element sensing the specific antigen-antibody binding reaction with a signal transduction system. The presence of a biological toxin is determined through the conversion of physico-chemical changes driven by an antibody-antigen (Ab/Ag) reaction in to analytical signals by TREIS technique. An application of novel nano-structured material for the sensor’s substrate has significantly improved its binding capacity, hence, sensor’s output. Performed electrochemical detection with non-polarized and polarized sensing electrodes allowed to determine the optimal parameters for the SEB toxin detection. The developed measuring method is capable of selectively separating the specific response in the real part of impedance to Ab/Ag reaction from the background effect of electrolytes as well as to distinguish the test sample, containing an extremely low concentration of SEB. Using the developed immobilization method and optimized detection regime, it is possible to determine in real time the presence of biological toxins in concentrations less than 10 pg mL^−1^.

## 4. Experimental Section

### 4.1. Materials

Oxalic acid (anhydrous 98%) and phosphoric acid (85% water solution) were purchased from Acros Organics (NJ, USA). Ethanol, HPLC grade and acetone, HPLC grade were obtained from Fisher Scientific (Fair Lawn, NJ, USA). Perchloric acid (60%) and 2-butoxyethanol [CH_3_(CH_2_)_3_OCH_2_CH_2_OH] were obtained from Alfa Aesar (Ward Hill, MA, USA) and J. T. Baker (Phillipsburg, NJ, USA). 3-Aminopropyltriethoxysilane (APTES) 98% was purchased from Strem Chemicals (Newburyport, MA, USA). Glutaraldehyde (70% v/v, Grade I), ethanolamine (98%), and sodium chloride (99%) were purchased from Sigma Aldrich (St. Louis, MO, USA). Electropolishing solution was prepared by mixing 70.0 vol.% of ethanol, 13.8 vol.% of distilled water, 10.0 vol.% of 2-butoxyethanol, and 6.2 vol.% of perchloric acid.

Monoclonal anti-*Staphylococcus aureus* enterotoxin B (anti-SEB) and *Staphylococcus aureus* enterotoxin B were purchased from Biodesign International (Saco, ME, USA) and Toxin Technology, Inc. (Sarasota, FL, USA), respectively. Sterilized deionized (SDI) water was used to prepare the reagents. Anti-SEB was dissolved with sterilized 0.01 M phosphate buffer saline (NaCl 0.08%) and stock solution was stored at −25 °C. SEB solution was prepared by diluting dissolving SEB and diluting with sterilized 0.3% NaCl solution (pH 6.0).

Commercial food-grade aluminum foil (alloy 1,100, thickness 0.25 mm), PTFE, polycarbonate, and stainless steel 316 were purchased from McMaster-Carr (Dayton, NJ, USA). To prepare sensor substrate blanks, aluminum foil was cleaned with acetone and cut into 12.8-mm discs.

### 4.2. Methods

#### 

##### Nano-patterning of the biosensor’s substrate: electropolishing and anodization

Nano-porous aluminum substrate for the biosensor was fabricated following a three-step procedure: annealing at 500 °C to homogenize surface structure via re-crystallization, electropolishing, and anodization. Electropolishing was performed as described elsewhere [19]. Aluminum discs were placed into the specimen holder and polished at 5 °C in electropolishing solution for 40 sec with vigorous stirring, under an applied voltage of 42 V, supplied by PC-controlled DC power supply, 1,787 A (BK Precision Corp., Yorba Linda, CA, USA). Electrochemical nano-patterning of the aluminum surface was performed by its anodization in 0.3 M oxalic acid solution. The overall process performance was monitored with multifunctional data acquisition system InstruNet 100 (InstruNet, Somerville, MA, USA). To obtain a well-ordered nano-porous surface structure the anodization was carried out at the temperature below 5 °C, controlled by the 3,016 Isotemp refrigerating circulator (Fisher Scientific, Pittsburgh, PA, USA). After the processing, aluminum discs were rinsed with deionized water and placed into 5 wt% phosphoric acid for 1 hr. Processed nano-porous aluminum discs were then washed with sterilized distilled (SDI) water and dried in nitrogen atmosphere. To prevent accidental surface contamination, prepared discs were stored in a sterilized vacuumed desiccator.

##### Sensibilization of nano-porous aluminum: silanization and anti-SEB immobilization

To immobilize antibodies, electrochemically processed aluminum discs were placed into a 2.5% v/v solution of APTES in ethanol for 4 hrs. Silanized aluminum surface was activated in the glutaraldehyde solution for 2 hrs. Then, activated aluminum discs were placed into the solution containing 40 μg·mL^−1^ of anti-SEB for 1 hr at 37 °C and, further, for 12 hrs at 4 °C. Remaining vacant sites on the sensor’s surface were blocked by soaking the discs in 100 mM ethanolamine solution for 1 hr. Completed anti-SEB sensing elements were thoroughly washed with SDI water, dried in nitrogen atmosphere, and stored at −25 °C.

##### Surface morphology control

The morphological changes of the aluminum surface due to electrochemical processing, APTES condensation, anti-SEB immobilization, and SEB conjugation were analyzed by scanning probe microscope (SPM) Q-Scope 350 (Quesant Inst. Corp., Agoura Hills, CA, USA) in tapping mode with NSC-16 cantilevers. A specialized software package, SPIP 3.3 (NanoScience, Phoenix, AZ, USA) was used for image processing and analysis of nanoscale patterns on aluminum surface.

##### Electrochemical measurements

Cyclic voltammogramms (CVA) and impedance spectra (IS) of anti-SEB immunosensor were collected with PC-controlled electrochemical workstation DHC2, equipped with PC4 (750)/DC105 potentiostat (Gamry Instruments, Warminster, PA, USA). Double junction Ag/AgCl reference electrode PHE 3211 (Omega Engineering, Inc., Stamford, CT, USA) was used for CV and EIS in the three-electrode setups. 0.3% NaCl stock solution, used in all experiments, was conditioned by purging high-pure nitrogen prior to the electrochemical measurements. CV scans were performed in the voltage range of −2.0...+1.0 V *vs.* Ag/AgCl reference electrode at the scan rate of 100 mVs^−1^. The impedance spectra were collected in the frequency range of 0.25 Hz …100 kHz with the excitation voltage of 10 mV. In the experiments with polarized working electrode, the sensor was polarized with Δ*φ*= +0.1 V *vs.* Ag/AgCl electrode. All presented experiments were triplicated. The error bars are placed on the plots where appropriate.

A custom-made electrochemical cell with two- and three- electrode configuration was used for all measurements. The cell contains a disk-shaped stainless steel counter electrode (CE), conductive support for the working electrode (sensor) embedded into the polycarbonate body with appropriate channels for filing and drainage of the testing solutions, and a placeholder for the reference electrode. This design allows to avoid interconnects on the sensor’s surface minimizing time and cost of the sensor fabrication and replacement. An internal compartment of the chamber is conically-shaped. Thus a counter electrode with larger surface area than that of a working electrode (*S_CE_*/*S_WE_* ∼ 10) can be used. This eliminates the effect of CE polarization on the sensor’s output.
